# Antibacterial activities of gold and silver nanoparticles against *Escherichia coli* and bacillus Calmette-Guérin

**DOI:** 10.1186/1477-3155-10-19

**Published:** 2012-05-06

**Authors:** Yan Zhou, Ying Kong, Subrata Kundu, Jeffrey D Cirillo, Hong Liang

**Affiliations:** 1 Materials Science and Engineering, Texas A&M University, College Station, TX, 77843, USA; 2 Department of Microbial and Molecular Pathogenesis, Texas A&M Health Science Center, Bryan, TX, 77807, USA; 3 Department of Mechanical Engineering, Texas A&M University, College Station, TX, 77843, USA; 4 Current address: ECMS Division, Central Electrochemical Research Institute, Tamilnadu, 630006, India

**Keywords:** Antibacterial effect, Gold, Silver, Nanoparticle, BCG

## Abstract

**Background:**

Diseases such as tuberculosis (TB) have always had a large impact on human health. Bacillus Calmette-Guérin (BCG) is used as a surrogate for TB during the development of anti-TB drugs. Nanoparticles (NPs) have attracted great interest in drug development. The purpose of this study was to examine the potential of NPs as anti-TB compounds by studying the interacting mechanisms between NPs and bacteria.

**Results:**

We investigated effects of gold and silver NPs on BCG and *Escherichia coli*. Experimentally, particle size and shape were characterized using transmission electron microscopy (TEM). Different concentrations of NPs were applied in bacterial culture. The growth of *E. coli* was monitored through colony forming units (CFU). The mechanism of interaction between NPs and bacteria was analyzed through bacterial thin sections followed by TEM and scanning electron microscopy. Antibacterial effects on BCG were observed by recording fluorescent protein expression levels.

**Conclusions:**

The results suggest NPs have potential applications as anti-TB compounds. The antibacterial effects and mechanism of action for NPs were dependent upon composition and surface modifications.

## Background

Diseases such as tuberculosis (TB) have always had a large impact on human health. According to a recent World Health Organization (WHO) report, there were an estimated 11.1 million prevalent cases and 9.4 million incident cases of TB in 2008. There are 1.3 million TB related deaths each year [1]. As a widely used TB vaccine, bacillus Calmette-Guérin (BCG) has been prepared from a strain of attenuated *Mycobacterium bovis,* which causes bovine tuberculosis. The virulence of BCG was lost after being cultured in potato medium for decades. In biosafety level 2 labs, it has been used as a surrogate for TB during the development of anti-TB drugs. Combination drug therapies are normally used against TB, since monotherapy fails to clear infections and leads to rapid development of resistance. There are drawbacks in current therapies, including drug-induced disease and the increasing prevalence of multiple-drug-resistant tuberculosis (MDR-TB). Nanoparticles (NPs) have attracted great interest in their development as potential antibacterial drugs [2,3]. It has been reported that biophysical interactions occur between NPs and bacteria including biosorption, NPs breakdown or aggregation, and cellular uptake, with effects including membrane damage and toxicity [4,5]. The mechanisms of NPs inhibiting bacterial growth remain less well understood. It has been reported that the size and surface modifications of NPs could affect their antibacterial levels [5,6]. Comprehensive understanding of antibacterial mechanisms is needed to improve the effectiveness of NPs in disease treatment.

Colloidal silver has been used as an antibacterial agent since ancient Greece [7]. Unlike antibiotic drugs, bacteria cannot easily develop resistance because silver targets multiple components in the bacterial cell. As a result, silver is used in medical equipment coatings [8] and dental resin components [9]. It is also reported that the mechanism behind its antibacterial activity is by weakening DNA replication and inactivating proteins [10]. On the contrary, gold has low toxicity to biological systems, whether bacteria, animal, or human, due to its elemental properties [11].

To date, comprehensive studies on nanoparticles have rarely been carried out in bacteria. To understand their interactions, we investigated the antibacterial effects of NPs with different compositions and surface modifications. Model bacteria *Escherichia coli* were tested with different NPs. Gold and silver NPs were chosen to have similar sizes and shapes. The growth of *E. coli* was evaluated by colony forming units (CFU). In order to investigate the mechanisms involved, transmission electron microscopy (TEM) and field emission scanning electron microscopy (FE-SEM) analyses were carried out. This mechanistic study provided information on how NPs interact with bacteria dynamically. The level of fluorescence expression was found to correlate with the numbers of viable BCG cells, as a result, we were able to monitor antibacterial activity of NPs on BCG cells. In terms of anti-TB drug development, this study suggests that NPs may represent useful candidates for therapeutics.

## Results

### Preparation and characterization of nanoparticles

**Figure 1:**
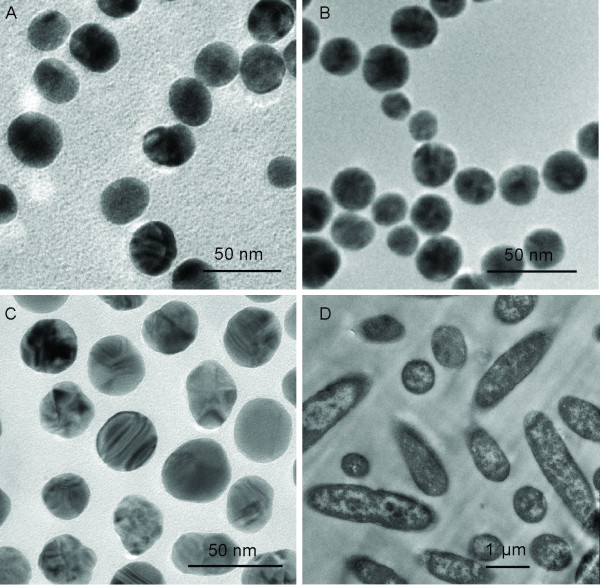
**TEM images of NPs.** Transmission electron micrographs of (A) citrate Au NPs after centrifugation and re-suspension in water, (B) PAH Au NPs as prepared, (C) Ag NPs after centrifugation and re-suspension in water, and (D) *E. coli* in the absence of NPs.

TEM micrographs of citrate Au and Ag NPs were obtained with NP solutions after centrifugation and re-suspension in DI water, and TEM micrographs of poly-allylamine hydrochloride (PAH) Au NPs were acquired with NPs in solution as they were made. In the case of Au NPs prepared in citrate, spherical NPs with 20−30 nm diameter were observed (Figure 1A), gold nanoparticles were well dispersed after rounds of centrifugation and re-suspension. PAH stabilized Au NPs were spherical and approximately 22 nm in diameter (Figure 1B), and the presence of polyelectrolyte forced the formation of monodispersed gold nanoparticle ‘aggregates’. Ag NPs were produced in Triton X-100 (TX-100) solution and reached an average diameter of 30 nm (Figure 1C). All NPs showed consistent spherical morphology and narrow size distribution between 20 and 30 nm. This consistency helped minimize the shape and size differences that might result in different antibacterial effects. Figure 1D shows thin section of *E. coli* grown in LB liquid medium. Bacterial cells exhibited well-defined cellular contents and were normal in size with intact intracellular structures.

**Figure 2:**
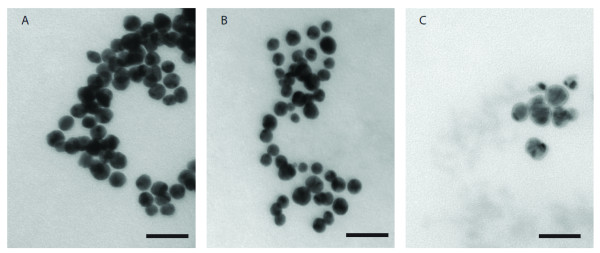
**NPs solution was added to LB medium for four hours to examine the aggregation.** (A) 10 μg/ml citrate Au NPs in LB medium. (B) 10 μg/ml PAH Au NPs in LB medium. (C) 10 μg/ml Ag NPs in LB medium. Scale bars represent 50 nm.

Nanoparticles in solution were added to fresh LB medium for four hours to examine the level of aggregation, as shown in Figure 2, and the final concentration of NPs were chosen to be 10 μg/ml. The level of aggregation of citrate Au NPs was higher in LB medium (Figure 2A) as compared to in DI water (Figure 1A). This might have been expected, since the medium is full of nutrients as well as free ions both of which could exchange with citrate on the nanoparticles surface and cause aggregation. The exchange process is less likely to occur when high molecular weight molecules, such as polyelectrolyte (PAH), were used for surface modifications. The result is the level of aggregation stayed the same after adding LB (Figure 2B). Ag NPs display aggregation after suspending them in LB medium (Figure 2C).

### Antibacterial effect of NPs on *E. coli*

**Figure 3:**
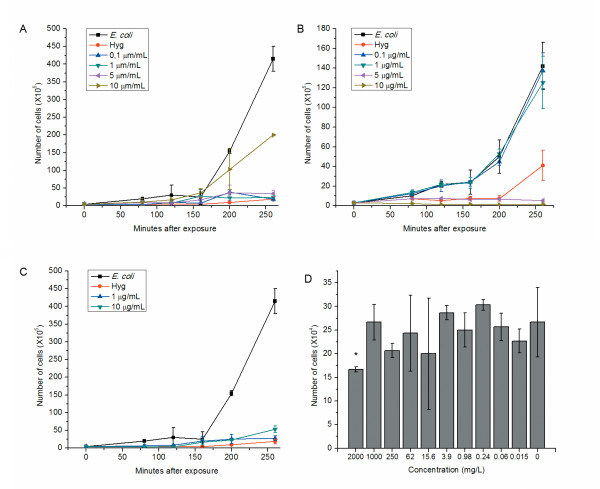
**The antibacterial effects of different NPs on*E. coli*and the MIC of PAH.** In the antibacterial test of NPs, bacterial cell cultures were set up at an initial optical density (OD) of 0.003 from an overnight culture. NPs solution was added into cell culture medium to reach predesigned concentrations. *E. coli* was exposed to 0.1, 1, 5, 10 μg/ml citrate and PAH Au NPs (A and B), and to 1, 10 μg/ml Ag NPs (C). 20 μg/ml hygromycin (Hyg) was used as a positive control and *E. coli* was used as a negative control. Experiment was repeated three times and the results are shown in mean ± SD. (D) The MIC of PAH against *E. coli*. Significant differences were observed on for the 2000 μg/ml PAH experimental group and the control group. *p<0.05.

NPs were added to LB medium to reach predesigned concentrations. *E. coli* was exposed to 0.1, 1, 5, 10 μg/ml citrate or PAH Au NPs and 1, 10 μg/ml Ag NPs. At selected times, 10 μl of medium was sampled, diluted and then cultured on LB ager plates. OD_600_ was also monitored over time. Considering the OD_600_ value of NPs in LB medium as background, it was expected that we could estimate bacterial numbers by subtracting background from each measurement. It was found that there was no OD_600_ additive relationship between NPs and bacteria (data not shown). After NPs were added to the bacterial culture, bacterial cells attracted NPs. Thus, the OD_600_ reading that would normally be generated by this portion of NPs was absent. As a result, CFU is likely to be the only accurate method to determine bacterial numbers with little influence from the NPs. CFU data were converted to bacterial numbers per ml as shown in Figure 3. The presence of NPs reduced the bacterial numbers during the exponential phase. High concentrations of citrate Au NPs have less of an inhibitory effect on bacterial growth as compared to low concentrations (Figure 3A). At 10 μg/ml, citrate Au NPs appeared to form aggregates which contain multiple mono-dispersed nanoparticles, and there is no opportunity for NPs inside ‘aggregates’ to interact with cells (Figure 2A). This also left fewer non-aggregated NPs which can be easily absorbed by bacteria. Hence, bacterial growth was only slightly inhibited at high citrate Au NPs concentrations. In contrast, *E. coli* growth was significantly inhibited by citrated Au NPs at 0.1, 1, 5 μg/ml. This observation could be explained by the fact that smaller aggregates exist at lower NPs concentrations. In such, more NPs can interact with bacterial cells and cause further damage. Figure 3B presents a dose-dependent relationship between PAH Au NPs and bacteria inhibition. It suggests that bacterial growth was less inhibited when 0.1 and 1 μg/ml PAH Au NPs were present, so there are similar growth curves compared to the negative controls. *E. coli* was largely inhibited by 5 and 10 μg/ml of PAH Au NPs. The effects of PAH toxicity on bacterial killing was evaluated by determining the minimal inhibitory concentration (MIC) for PAH. The initial concentration of PAH for making PAH Au NPs was 1000 mg/L, and most of PAH was removed after NPs formation by centrifugation and re-suspension in water. As shown in Figure 3D, the only concentration of PAH that display bacterial inhibitory effects is 2000 mg/L and lower concentrations showed no inhibitory effect. Hence, the antibacterial effect of PAH Au NPs is not due to concentrations of PAH that could remain after processing. At 260 min, hygromycin treated *E. coli* started to replicate while 5 and 10 μg/ml PAH Au NP treated groups still displayed no growth. This suggests that there are stronger antibacterial effects at these concentrations as compared to 20 μg/ml of hygromicin. Strong antibacterial activities were observed at all Ag NP concentrations (Figure 3C).

### Electron microscopic analysis

**Figure 4:**
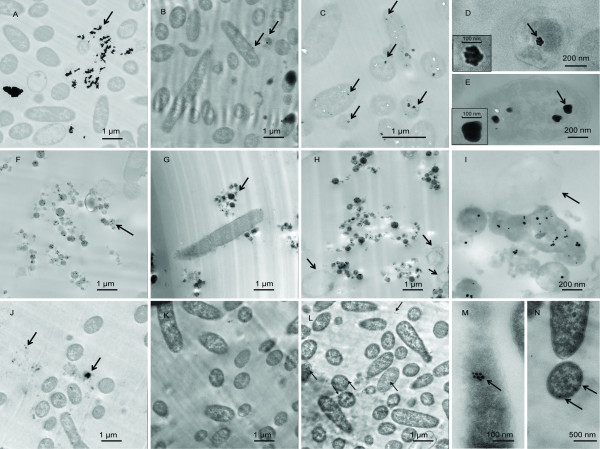
**TEM images of*E. coli*with NPs at different time points.** (A−E) *E. coli* with citrate Au NPs at (A) 3 h, (B) and (D) 6 h, (C) and (E) 9 h.(Inset) Magnified 100 nm complex pointed by arrows. (F−I) *E. coli* with PAH Au NPs at (F) 3 h, (G) 6 h, (H) and (I) 9 h.(J−N) *E. coli* with Ag NPs at (J) 3 h, (K) and (M) 6 h, (L) and (N) 9 h. Arrows point to NPs (A−G, J−N) and hollow cells (H and I).

Nanopaticle treated *E. coli* was thin sectioned for TEM imaging in order to study the mechanisms of the antibacterial interactions. The following concentrations were selected because they display strong antibacterial effects: 2 μg/ml citrate Au NPs, 5 μg/ml PAH Au NPs and 20 μg/ml Ag NPs. Samples were withdrawn and fixed at 3, 6, and 9 hours. These points were picked since the interaction is more obvious. Seen from TEM micrographs, NPs are rarely found within *E. coli* cells after 3 hours of incubation with citrate Au NPs (Figure 4A, arrow indicates Au NPs aggregates). Most of the NPs are either free in the medium or aggregated between bacteria. Bacterial cell walls started to retain some NPs at 6 hours (arrows in Figure 4B). Figure 4C demonstrates an increased number of NPs inside bacterial cells after 9 hours (arrows in Figure 4C). The NP complexes inside bacterial cells in Figure 4D and 4E were measured to be 100 nm in diameter, which is equivalent to about 50 individual NPs and their morphology is quite different from more dispersed ‘aggregates’ in Figure 2A. It is easy to distinguish a single NP within the 100 nm complex at 6 hours (inset in Figure 4D). However, given more time, the outlines of individual NP became blurry within these complexes, which suggests that single NPs physically merged with each other and ‘aggregates’ turned into larger particles (9 h; inset in Figure 4E). PAH Au NPs showed different antibacterial activity when they were applied in *E. coli* culture, cell lysis was observed at 3 h, 6 h, and 9 h (Figure 4F−4I). Au NPs were trapped within what appeared to be released cytoplasm and no free NPs were found in the medium (arrows in Figure 4F and 4G). Release of the cytoplasm left hollow and disfigured cell walls (arrows in Figure 4H and 4I). TEM micrographs of Ag NPs treated *E. coli* at different time points are shown in Figure 4J−4N. Arrows in Figure 4J indicate Ag aggregates formed in medium, there are no aggregated Ag NPs complexes inside of cells. As seen in Figure 4M and 4N, NPs maintain their original size and spherical shape. A large portion of the Ag NPs was trapped within the cell walls (Figure 4L−4N).

**Figure 5:**
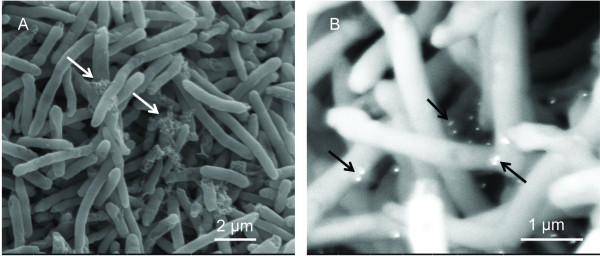
**FE-SEM of*E. coli *in the presence of citrate Au NPs.** (A) secondary electron image, (B) backscattered electron image. Arrows point to Au NPs

FE-SEM was used to examine the interactions of NPs with bacteria in more detail and the results are shown in Figure 5. *E. coli* was fixed after 30 min of incubation with citrate Au NPs. In secondary electron mode, Au NPs were found aggregated (Figure 5A), which is in accordance with the TEM result in Figure 3A. Besides aggregated Au NPs, some Au NPs were found dispersed among cells, as shown through back-scattered imaging (Figure 5B).

### Antibacterial effect of NPs on BCG

**Figure 6:**
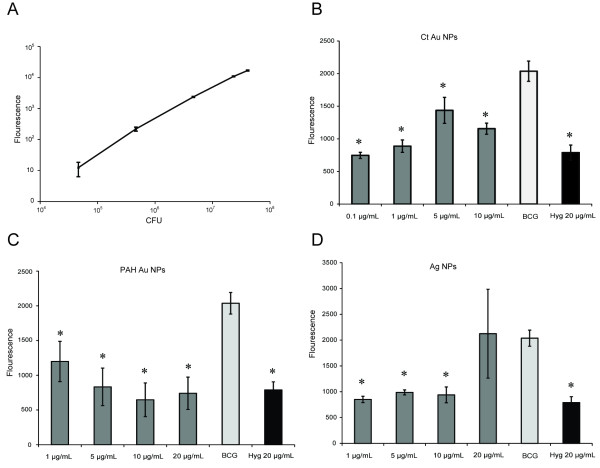
**Fluorescence expression of BCG.** Tdtomato fluorescence protein expressed by bacillus Calmette-Guérin (BCG) allows fast detection of viable bacteria cells under treatment with NPs. (A) Correlation of the number of BCG and fluorescence level was determined by CFU, R^2^ = 0.994. At day zero, fluorescence of BCG was 701.7 ± 61.6. After five days incubation with NPs, (B) Fluorescence of BCG with 0.1, 1, 5, 10 μg/ml citrate Au NPs. (C) Fluorescence of BCG with 1, 5, 10, 20 μg/ml PAH Au NPs. (D) Fluorescence of BCG with 1, 5, 10, 20 μg/ml Ag NPs. The experiment was repeated three times and the results are shown in mean ± SD. Comparison was made with NP-free BCG control to determine the antibacterial effects. *p<0.05: significant difference from NP-free BCG control.

The relationship between viable cell numbers and fluorescence was shown in Figure 6A. The level of fluorescence was correlated with bacterial numbers, as confirmed by CFU (R^2^ = 0.994). All NPs were found to have no emission at 554 nm excitation. Hence, there is no interference of NPs with fluorescence emission. After 5 days of BCG/NPs co-culture, fluorescence was measured and the results are shown in Figure 6B−6D. BCG fluorescence was inhibited by citrate Au NPs at all concentrations tested (Figure 6B). The inhibitory effect of citrate Au NPs was more obvious at 0.1 and 1 μg/ml which is in accordance with *E. coli* inhibition. Low fluorescence levels were found when PAH Au NPs were at high concentrations (Figure 6C). This dose-dependent relationship for PAH Au NPs was observed for both *E. coli* and BCG. When BCG was treated with 1, 5, 10 μg/ml of Ag NPs, it displayed similar fluorescence to that of the hygromycin treated group ( 6D).

## Discussion

**Figure 7:**
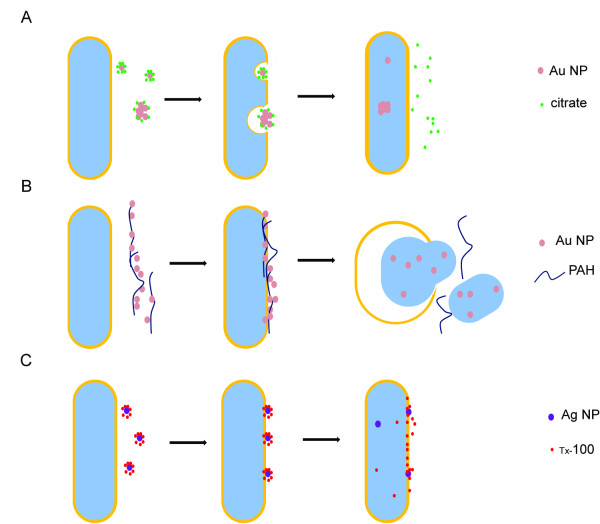
**Schematic representation of the interactions between NPs and bacterial cells.** (A) Bacterial cells take up single citrate Au NPs or aggregations of Au NPs complexes. (B) PAH facilitates Au NPs uptake into bacterial cells followed by lysis. (C) Most of Ag NPs were trapped in cell walls.

In the present research, we evaluated the antibacterial activity of nanoparticles. These particles differ in combinations of composition, types, and surface modifications while their size and shape remain the same. The surface charge and chemical properties of NPs are determined by capping agents, which play an important role during NPs and bacterial interactions. For the same type, NPs stabilized with a weakly bound capping agent (citrate) tend to aggregate more as compared to those with a strongly bound capping agent (PAH), as shown in Figure 1A and 1B. The increased aggregation means reduced the surface area that would reduce the interactions between NP and bacteria resulting bacterial inhibitory interactions. The evidence is seen in non-dose dependent relations (Figure 3A). Citrate has been used as a carbon source to differentiate *E. coli* from other species since the bacteria is incapable of transferring citrate through its membrane [12,13]. The formation of aggregates is most likely caused by citrate reduction within the capping agent [14]. It is believed that ion/molecule in medium exchange with citrate resulted in this reduction and thus formed ‘loose’ aggregates in medium. Since exchange is heterogeneous and some Au NPs remained the original 20 nm in size, once they have entered a cell, Au NP aggregation turned into complex structures where single NP physically connected with each other while citrate ions were kept outside the cell. These are seen in Figure 4D and 4E. Bacteria appeared to take up single nanoparticles and rearrange them inside cytoplasm, as shown in Figure 4C and 4E, the particles within cells showed a broad size range. The proposed mechanism is described in Figure 7A. Due to the increased size and resistance to cutting resulted of NP complexity, dislocation appeared when the sectioning blade was in contact with the NPs during sample sectioning. Holes observed in the sections represent the positions where NPs were located prior to sectioning (Figure 4C).

Our results suggest a different antibacterial mechanism for PAH Au NPs from citrate Au NPs. A previous report shows that PAH Au NPs self-assemble into 4−5 micron long chains, which is an indication of strong interactions between NPs and PAH [15]. As a result, PAH NPs did not further aggregate in culture media and dose-dependent relations seen in Figure 3B. Furthermore, PAH organized chain-like aggregates were disturbed once they entered the cell, and more scattered Au NPs were found within pools of released cytoplasm (Figure 4F−4I). The hypothesis for this mechanism is shown in Figure 7B. PAH facilitated the delivery of a large number of Au NPs that strongly bond to PAH on the bacterial cell surface. Bacteria cell wall tends to attract positive charged PAH due to the total charge of cell wall being negative [16]. As a result, cell walls encounter high stress as they accumulate PAH and Au NPs. Since the MIC of PAH is well above the concentrations likely to be present in the NPs, as shown in Figure 3D, the PAH is not responsible for the toxic effects of NPs for bacteria. Once Au NPs penetrate the cell wall and enter the cytoplasm, PAH is more likely to have direct contact with the cell membrane through damaged cell wall. Then, PAH could play a role in accelerating cell wall breakdown and cytoplasm release. It has been reported that cationic coated Au NPs are more toxic than anionic coated ones [16], and this supports the concept that PAH Au NPs caused immediate cell lysis while citrate Au NPs did not. Furthermore, the mechanism of interaction between Ag NPs and *E. coli* is described in Figure 7C. The high antibacterial activity resulted from the nature of the silver element, and possibly the neutral TX-100, which could participate in the interaction. Based on FE-SEM images, several Au NPs were very close to each other tending to aggregate (arrows in Figure 5B). NPs tend to bind to the long end of the cell, it is likely that the ends providing a better site for attachment.

In antibacterial tests against BCG, NPs successfully reduced BCG’s fluorescence. Since the level of fluorescence correlates with numbers of viable BCG as tested by CFU ( 6A), this assay provides a fast way to monitor the growth of slow-glowing mycobacteria. From the reduction of fluorescence, we can conclude that BCG growth is inhibited by NPs, which could be ideal candidates for drug development.

## Conclusions

In conclusion, we have demonstrated that gold and silver nanoparticles display excellent antibacterial potential for the Gram negative bacteria *E. coli* and the Gram positive bacteria BCG*.* These NPs display their best performance when aggregation is not observed at high levels. By changing surface modifications agents, gold NPs with the same shape and size exhibited different inhibitory effects. Our mechanistic analyses indicated that PAH capped gold NPs caused cell lysis, while citrate capped gold NPs did not. Strong antibacterial activities were observed for silver NPs due to their inherent elemental properties. In terms of anti-TB drug development, this study suggests that NPs may represent useful candidates, but will require significant development to ensure optimal bactericidal activity and low host toxicity.

## Methods

### Nanoparticles (NPs)

Citrate stabilized gold (Au) NPs were prepared following previously reported methods [17]. Citrate ions produce negative charges on the surface of nanoparticles. By controlling citrate and hydrogen tetrachloroaurate tri-hydrate (HAuCl_4_) concentrations, 20−30 nm diameter Au NPs were synthesized. Poly-allylamine hydrochloride (PAH) stabilized Au NPs with 22 nm mean size were synthesized in an aqueous solution of HAuCl_4_ and PAH and in the presence of gold seed particles [15]. A positive charge was demonstrated on Au NPs’ surface because of the presence of PAH. Silver (Ag) NPs were produced by photoirradiating AgNO_3_ in Triton X-100 (TX-100) solution for 60 min, the average size was around 30 nm [18]. Citrate Au NPs and Ag NPs solutions were centrifuged at 8000 rpm for 15 min to remove extra citrate and TX-100. The precipitated NPs were suspended in DI water. This process was repeated 3−4 times. PAH Au NPs were used immediately after they were made, since precipitated NPs were unable to dissolve in water.

### Bacterial Growth and Exposure

pBlueScriptKSII + plasmid (Agilent Technologies, Santa Clara, CA) was transformed into *Escherichia coli*DH5α (Invitrogen, Carlsbad, CA). *E. coli* was grown in liquid Luria-Bertani (LB) medium at 37°C and 250 rpm. Bacillus Calmette-Guérin (Pasteur) cultures expressing tdTomato fluorescent protein were grown in M-OADC-TW broth with Middlebrook (M) 7 H9 broth (Difco) supplemented with 10% (*v/v*) oleic acid albumin dextrose complex, 0.5% (*v/v*) glycerol, and 0.05% (*v/v*) Tween 80. Medium was supplemented with 25 μg/ml kanamycin. NPs in solution were added into bacteria cultures to reach specific concentrations.

### Characterization

NPs samples for TEM imaging were prepared by slow evaporation of freshly made NPs in solution on a carbon-coated copper grid at room temperature. Bacterial samples were fixed in 2% (*v/v*) glutaraldehyde for 0.5 h at 37°C by adding fixative into culture medium. The following process was performed with cold microwave technology in the BioWave. The microwave was set for a 6 minute cycle (2 min power on, 2 min power off, 2 min power on) at 200 W for primary fixation. Alternating vacuum cycles of 30 seconds were used during 6 minutes of fixation. The microwave temperature was set at 20°C for all steps. Bacteria were spun down and washed 3 times with 0.1 M HEPES for 1 min at 200 W with consistent vacuum. Subsequently, 1% (*v/v*) osmium tetroxide in HEPES buffer was used for overnight post fixation. Samples were microwaved for 6 min at 100 W at the same power and vacuum cycles as used in primary fixation. Methyl alcohol was used for dehydration in 10% (*v/v*) steps from 10% to 100%. Microwave was set at 100 W with 1 min per step. Quetol 651 epoxy resin was used for specimen embedding. Thin sections were picked up on 200 mesh copper grids and imaged with a JEOL 1200EX TEM with 100 keV acceleration voltage [19]. FE-SEM sample preparation followed the same fixation and dehydration procedure described above. Images were taken on an FEI Quanta 600 FE-SEM at an acceleration voltage of 10 keV. ImageJ software was used for measuring nanoparticle size distribution.

### Antibacterial test

*E. coli* cells were grown in LB liquid medium at 37°C for 12 hours before they were diluted in fresh LB liquid medium to reach OD_600_ = 0.003 (optical density). Gradient concentrations of NPs were then added to the culture medium. Bacteria/NP mixed cultures were put into a 37°C incubator. At different time points of 80, 120, 160, 200, and 260 min the medium was withdrawn from each sample. Dilutions were then made and cultured on LB agar plates. Plates were incubated overnight at 37°C and CFU was determined. NP-free *E. coli* was used as a negative control and 20 μg/ml hygromycin was used as a positive control for bactericidal activity. The toxicity of PAH was tested by determining its minimum inhibitory concentration (MIC). Overnight cultured *E. coli* suspensions were adjusted in fresh LB liquid medium to reach an OD_600_ = 0.1 and diluted by a factor of 1:100. PAH was dissolved in LB media. After mixing 50 μL *E. coli* dilution and 50 μL PAH solution, 10 μL of the sample was immediately drawn out and a series of dilutions made. Samples were plated on LB agar plates, and colonies were counted the next day [20].

### Fluorescence Spectrophotometer

BCG::tdTomato with OD_600_ = 0.05 was cultured in 96 well plates with different NPs at various concentrations. Five days after mixing, fluorescence was measured using a Perkin Elmer Envision spectrophotometer with an excitation wavelength of 554 nm and emission wavelength of 581 nm. Wells with NP-free BCG::tdTomato were used as negative controls, and the wells with 20 μg/ml hygromycin in NP-free 7 H9 medium were used as positive controls for comparison. The *t* test (student version) was used to determine the significance of results.

## Abbreviations

BCG = Bacillus Calmette-Guérin; CFU = Colony forming units; FE-SEM = Field emission scanning electron microscopy; Hyg = Hygromycin; LB = Luria-Bertani; MIC = Minimum inhibitory concentration; NP = Nanoparticle; OD600 = Optical density at 600 nm; PAH = Poly-allylamine hydrochloride; TB = Tuberculosis; TEM = Transmission electron microscope; TX-100 = Triton X-100.

## Competing interests

The authors declare no competing interests.

## Authors’ contributions

YZ designed the experiments, performed the bacterial culture, counted CFU, sectioned bacteria, performed TEM and FE-SEM observation on bacteria, did fluorescence examination, and drafted manuscript. YK maintained bacterial strains. SK prepared NPs and characterized NPs. JDC and HL conceived research, participated in experimental design, and participated in manuscript writing. All authors read and approved the final manuscript.
